# Development of a convolutional neural network-based AI-assisted multi-task colonoscopy withdrawal quality control system (with video)

**DOI:** 10.3389/fphys.2025.1666311

**Published:** 2025-10-09

**Authors:** Jian Chen, Menglin Zhu, Zhijia Shen, Kaijian Xia, Xiaodan Xu, Ganhong Wang

**Affiliations:** ^1^ Department of Gastroenterology, Changshu Hospital Affiliated to Soochow University, Suzhou, Jiangsu, China; ^2^ Center of Intelligent Medical Technology Research, Changshu Hospital Affiliated to Soochow University, Suzhou, Jiangsu, China; ^3^ Department of Nursing, Changshu Hospital Affiliated to Nanjing University of Chinese Medicine, Suzhou, Jiangsu, China; ^4^ Department of Gastroenterology, Changshu Hospital Affiliated to Nanjing University of Chinese Medicine, Suzhou, Jiangsu, China

**Keywords:** artificial intelligence, YOLO, colonoscopy, effective withdrawal time, colonoscope withdrawal speed, blurdetection

## Abstract

Background Colonoscopy is a crucial method for the screening and diagnosis of colorectal cancer, with the withdrawal phase directly impacting the adequacy of mucosal inspection and the detection rate of lesions. This study establishes a convolutional neural network-based artificial intelligence system for multitask withdrawal quality control, encompassing monitoring of withdrawal speed, total withdrawal time, and effective withdrawal time. Methods This study integrated colonoscopy images and video data from three medical centers, annotated into three categories: ileocecal part, instrument operation, and normal mucosa. The model was built upon the pre-trained YOLOv11 series networks, employing transfer learning and fine-tuning strategies. Evaluation metrics included accuracy, precision, sensitivity, and the area under the curve (AUC). Based on the best-performing model, the Laplacian operator was applied to automatically identify and eliminate blurred frames, while a perceptual hash algorithm was utilized to monitor withdrawal speed in real time. Ultimately, a multitask withdrawal quality control system—EWT-SpeedNet—was developed, and its effectiveness was preliminarily validated through human-machine comparison experiments. Results Among the four YOLOv11 models, YOLOv11 m demonstrated the best performance, achieving an accuracy of 96.00% and a precision of 96.38% on the validation set, both surpassing those of the other models. On the test set, its weighted average precision, sensitivity, specificity, F1 score, accuracy, and AUC reached 96.58%, 96.44%, 97.64%, 96.38%, 96.44%, and 0.9975, respectively, with an inference speed of 86.78 FPS. Grad-CAM visualizations revealed that the model accurately focused on key mucosal features. In human-machine comparison experiments involving 48 colonoscopy videos, the AI system exhibited a high degree of consistency with expert endoscopists in measuring EWT (ICC = 0.969, 95% CI: 0.941–0.984; r = 0.972, p < 0.001), though with a slight underestimation (Bias = −11.1 s, 95% LoA: −70.5 to 48.3 s). Conclusion The EWT-SpeedNet withdrawal quality control system we developed enables real-time visualization of withdrawal speed during colonoscopy and automatically calculates both the total and effective withdrawal times, thereby supporting standardized and efficient procedure monitoring.

## 1 Introduction

Colorectal cancer (CRC) is the third most common malignancy worldwide, with approximately 1.93 million new cases and 930,000 deaths reported globally in 2020. These numbers are projected to rise to 3.2 million new cases and 1.6 million deaths by 2040 (Morg et al., 2023). As the gold standard for the diagnosis and screening of colorectal diseases, the quality of colonoscopy procedures has a direct impact on the adenoma detection rate (ADR) and the risk of interval colorectal cancer (Hsu and Chiu, 2023).

In the colonoscopy procedure, quality control (QC) during the withdrawal phase is regarded as a critical component. Studies have demonstrated that prolonging the withdrawal time (WT) and ensuring comprehensive and stable mucosal inspection during withdrawal can significantly enhance the adenoma detection rate (ADR) and reduce the risk of missed diagnoses (Rex et al., 2024). However, in clinical practice, interventional procedures such as biopsies and polypectomies frequently occur during withdrawal, consuming a portion of the examination time—this is referred to as the “negative colonoscopy withdrawal time.” Additionally, certain procedural behaviors, including bowel irrigation, close contact between the endoscope and the intestinal wall leading to darkened screens, or blurred frames caused by rapid lens movement, may disrupt mucosal visualization and produce non-interpretable “blur frames.” Although these ineffective frames are counted in the total WT, they do not contribute meaningfully to mucosal assessment. Therefore, compared with WT, a more precise indicator of mucosal observation quality is the effective withdrawal time (EWT)—the actual time spent on mucosal inspection, excluding periods of interventional manipulation and blur frames. A study by Lui et al. (2024) found that EWT calculated by artificial intelligence had a stronger correlation with ADR, with each additional minute of EWT associated with a 49% increase in ADR. Moreover, the consistency of withdrawal speed is another key metric for evaluating withdrawal quality. Excessive speed may lead to incomplete visualization of the mucosa and an increased risk of missed lesions (Gong et al., 2023); conversely, even if the total recommended withdrawal time is met, large fluctuations in withdrawal speed can compromise the continuity and quality of mucosal observation.

In recent years, artificial intelligence (AI) technologies have shown tremendous potential in enhancing quality control (QC) during colonoscopy procedures (Chen et al., 2024). Previous studies (De Carvalho et al., 2023) have explored the use of AI to automatically identify the cecal landmark and measure withdrawal duration, thereby providing an objective assessment of whether the standard withdrawal time has been achieved and enabling real-time procedural intervention. However, to date, no study has systematically realized the simultaneous and real-time monitoring of withdrawal time (WT), effective withdrawal time (EWT), and withdrawal speed.

Therefore, this study aims to develop an AI-assisted, multitask colonoscopy withdrawal quality control system based on convolutional neural networks (CNN). The system is designed to achieve real-time, synchronized monitoring and visual feedback of withdrawal speed, WT, and EWT, thereby promoting procedural standardization, optimizing workflow, and ultimately improving adenoma detection rates while reducing the risk of missed colorectal cancers.

## 2 Methods

### 2.1 Study design and datasets

**FIGURE 1:**
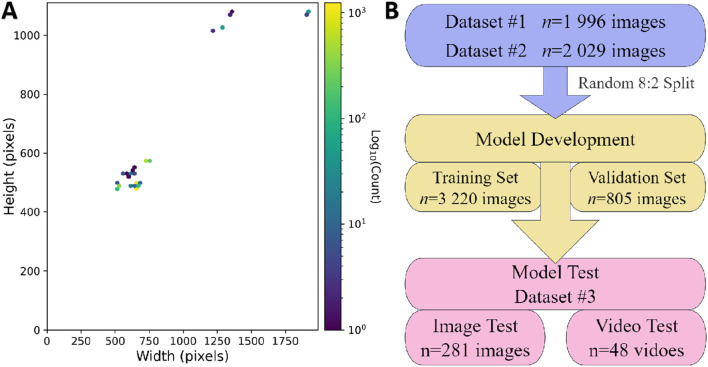
Dataset characteristics analysis. **(A)** Distribution of image resolutions: Yellow indicates a higher frequency of images with a specific resolution, while purple denotes lower frequency. The dataset includes images of various sizes, with the two most common resolutions being 664 × 479 pixels and 660 × 497 pixels. **(B)** Dataset partition overview.

**FIGURE 2:**
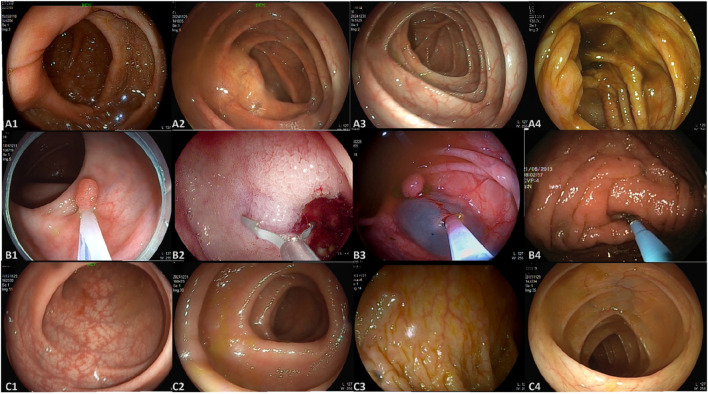
Representative images from the dataset. **(A)** A1–A4 represent images of the ileocecal part; **(B)** B1–B4 depict various instrument operations; **(C)** C1–C4 show normal visible mucosa.

This study utilized three datasets spanning from January 2020 to February 2025, comprising a total of 4,025 colonoscopy images and 48 videos. Dataset 1 and Dataset 2 were collected from Changshu Hospital Affiliated to Soochow University and Changshu Shanghu Central Hospital, respectively, containing 3,744 colonoscopy images used for model training and validation. Dataset 3, provided by Changshu Hospital of Traditional Chinese Medicine, included 281 images and 48 colonoscopy videos, serving as the external test set. To ensure its independence, the test set was used solely for performance evaluation and was not involved in model training or parameter tuning. An overview of dataset characteristics is presented in Figure 1. The image categories within the datasets were annotated into three classes: ileocecal part, instrument operations, and normal visible mucosa, with representative examples shown in Figure 2. The three participating medical centers employed colonoscopy equipment from three different manufacturers: six Olympus systems (OLYMPUS CV-V1, Japan Olympus Corporation), four SonoScape systems (HD-550, SonoScape Medical Corp., Shenzhen, China), and two Pentax systems (EPK-i7000, Pentax Medical, a division of HOYA Group, Japan).

### 2.2 Image annotation

**FIGURE 3:**
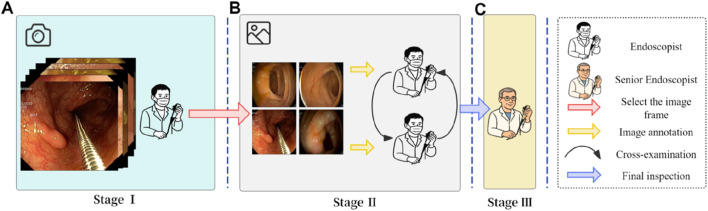
Image annotation workflow. **(A)** Stage I: Endoscopists selected representative colonoscopy video segments and converted them into individual image frames. **(B)** Stage II: Two independent teams of endoscopists screened the frames and retained only clear images with identifiable features. **(C)** Stage III: A senior endoscopist reviewed and finalized the annotations to ensure consistency and accuracy.

The image annotation process in this study was conducted in three stages (Figure 3), with each stage assigned to a distinct team of endoscopists. Prior to annotation, all team members underwent multiple rounds of theoretical training and hands-on practice related to the project to ensure annotation quality and consistency. Stage I involved the selection of video segments by endoscopists, which were then converted into individual image frames. In Stage II, two independent teams of endoscopists screened the images, retaining only clear frames containing various types of lesions, followed by cross-validation. Stage III was conducted by senior endoscopist, who reviewed the annotation results and made the final determinations. The annotators involved in the labeling process had varying levels of clinical experience. Specifically, the endoscopists in Stage I and II had 2–5 years of experience in gastrointestinal endoscopy, while the senior endoscopist in Stage III had over 10 years of clinical experience.

### 2.3 Model training configuration

This study adopted a transfer learning strategy (Shin et al., 2016), utilizing four variants of the YOLOv11 model (nano, small, medium, and large) pre-trained on ImageNet (Khanam and Hussain, 2024), and fine-tuned all layers using the study-specific dataset. The training configuration included automatic optimizer selection, dynamic learning rate adjustment, a maximum of 100 training epochs, and a batch size of 32. Evaluation metrics comprised accuracy, precision, recall, and F1 score. Training was accelerated using automatic mixed precision on GPU devices, with an early stopping strategy (patience = 8) employed to prevent overfitting. All training procedures were implemented using the PyTorch framework. To ensure model robustness and practical applicability, the performance of the four YOLOv11 variants was compared, and the best-performing model was selected.

The selection of YOLOv11 was based on its strong performance, technical maturity, and suitability for real-time medical image analysis. The model achieves an excellent balance between accuracy and inference speed, making it ideal for video-based colonoscopy tasks. Its modular architecture and well-established training pipeline also facilitate integration into multitask systems like ours, especially in clinical environments with limited programming resources. In addition, our team has previously applied YOLOv11 in another published study focused on auricular acupoint keypoint detection, where it also demonstrated robust and accurate performance (Wang et al., 2025) (Qiu et al., 2023), further confirming its versatility across medical AI applications.

To enhance the model’s generalization capability, this study implemented a series of image preprocessing and data augmentation strategies. During preprocessing, all images were uniformly resized to 640 × 640 pixels while preserving the original aspect ratio. The data augmentation techniques included: (1) random horizontal flipping with a 50% probability; (2) random resizing and cropping (RandomResize, RandomCrop); and (3) HSV-based random perturbation using YOLO’s HSVRandomAug algorithm to improve robustness to variations in lighting and color (Li et al., 2022). All augmentation operations were performed online in real time (Zhang et al., 2021), eliminating the need for additional image storage and ensuring that the model was exposed to diverse variations in each training iteration.

### 2.4 Development of the multitask withdrawal quality control system

#### 2.4.1 Definition of effective withdrawal time

Withdrawal time (WT) is defined as the duration from the moment the colonoscope reaches the ileocecal region (specifically, the ileocecal valve at the terminal ileum) to its complete withdrawal through the anus. WT primarily includes two segments of non-observational time: Time 1 refers to the duration of instrumental operations, such as chromoendoscopy, biopsy, and polypectomy procedures (e.g., endoscopic mucosal resection and cold snare polypectomy); Time 2 denotes periods of non-interpretable frames, including bowel irrigation, darkened screens, or blurred images caused by rapid scope movement. After excluding these ineffective periods, the remaining duration constitutes the actual time used by the endoscopist to inspect the mucosa and detect lesions, known as the Effective Withdrawal Time (EWT). The theoretical formula is: EWT = WT − Time 1 − Time 2.

#### 2.4.2 AI-Based automatic calculation of EWT

To enable the automated AI-based calculation of EWT, this study developed a multitask classification model based on a convolutional neural network (YOLOv11). The workflow is as follows: the system processes colonoscopy videos frame by frame, using the model to identify each frame as one of three categories in real time—ileocecal part, instrumental operation, or normal mucosal observation. When a sequence of ileocecal frames is detected, the system automatically marks the beginning of withdrawal and sets that frame as the starting point of WT.

The model then continuously monitors subsequent frames to detect instrumental operation frames (Time 1). Simultaneously, to identify blurred frames within non-observational footage (Time 2), a Laplacian operator is employed for image clarity assessment. This method evaluates image sharpness by calculating the second-order derivative of the grayscale image and determining its variance, using the formula: Var_*L*_ = Var(*∇* ^*2*^ *I*), where *∇* ^*2*^ *I* represents the Laplacian response of image *I*, and Var denotes variance. If Var_*L*_ is less than 50, the frame is classified as blurred and counted toward Time 2. The system accumulates the frame counts corresponding to Time 1 and Time 2 in real time, subtracts them from the total WT, and converts the result into EWT based on the frame rate. This approach enables the automatic and real-time computation of key quality control indicators during the colonoscopy withdrawal process, eliminating the need for manual intervention.

#### 2.4.3 Automated monitoring of withdrawal speed

To monitor withdrawal speed during colonoscopy, this study employed the perceptual hash (pHash) algorithm to quantify visual differences between consecutive video frames. Specifically, the pHash value of each frame *I* was calculated using the imagehash library, denoted as: *H* = pHash (*I*). The hash difference between adjacent frames was measured by computing the Hamming distance, defined as: *D* = *H1* - *H2*, where *H1 and H2* represent the pHash values of two consecutive frames. The resulting difference *D* reflects the degree of visual content change and serves as an indirect indicator of the colonoscope’s movement speed. To enhance visualization, a speed scale bar was overlaid on each video frame, displaying the current speed status based on the hash difference D. The system used a color-coded scheme: blue for normal speed (*D* ≤ 20), yellow for warning speed (21 ≤ *D* ≤ 30), and red for hazardous speed (*D* > 30). This approach enables intuitive, real-time speed monitoring without relying on positional tracking or external sensors, thereby supporting operational stability and improving mucosal observation quality during withdrawal.

#### 2.4.4 System integration and functionality implementation

**FIGURE 4:**
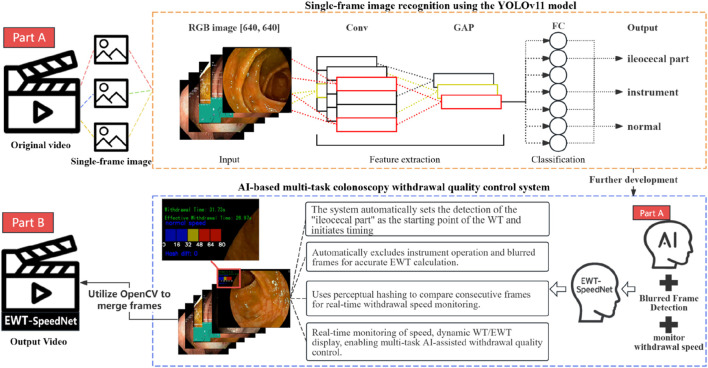
Schematic architecture of the multitask colonoscopy withdrawal quality control system.

Building upon the aforementioned modules, this study developed an integrated AI-assisted multitask colonoscopy withdrawal quality control system, named EWT-SpeedNet, with its system architecture illustrated in Figure 4. The system utilizes the YOLOv11 model to perform real-time image classification, automatically identifying the withdrawal start point while simultaneously calculating WT, Time 1, Time 2, and EWT. In addition, by incorporating perceptual hash (pHash) and Hamming distance analysis, the system quantitatively evaluates variations in withdrawal speed, which are visualized using a color-graded scale bar. Constructed using PyTorch and OpenCV, the system enables real-time display of multiple quality control metrics—including WT, EWT, and withdrawal speed monitoring—within a unified, user-friendly interface. Requiring neither additional hardware nor manual input, EWT-SpeedNet offers continuous, objective, and efficient real-time feedback to support clinical practice.

### 2.5 Model interpretability analysis

To enhance model transparency, this study employed explainable artificial intelligence techniques by using Grad-CAM to generate heatmaps (Barclay et al., 2006), visually illustrating the regions of the image the model focuses on during decision-making. The process involves defining a model wrapper, *MyModelWrapper*, to adapt the YOLOv11 output, selecting the penultimate layer as the target for visualization, and initializing Grad-CAM after loading and transferring the model to the GPU.

Images are then read, converted to RGB, resized to 224 × 224 pixels, normalized, and transformed into tensors with gradient tracking enabled. After model inference, a grayscale heatmap is generated and superimposed on the original image. The original image, heatmap, and final visualization are saved for analysis. These visual outputs allow researchers and clinicians to verify whether the model focuses on clinically relevant anatomical or pathological areas, which not only enhances interpretability but also fosters trust in the model’s decisions. Such transparency is crucial in clinical environments, where explainability strongly influences the adoption of AI tools in routine practice.

### 2.6 Human–AI comparison

This study systematically compared the performance of the AI system (AI group) with two teams of human endoscopists (junior group and senior group) in measuring EWT/WT. The design and methodology are as follows: A total of 48 colonoscopy videos, labeled 1–48, were selected. All videos were anonymized, randomly coded, and distributed to each physician and the AI system. The junior group consisted of three endoscopists with 0–5 years of experience, while the senior group included three endoscopists with more than 10 years of experience. Summary statistics: The EWT and WT of all 48 videos were calculated for both the junior and senior groups. Consistency assessment: The intraclass correlation coefficient (ICC) was used to evaluate the consistency between the AI system and the mean values of the senior group, using a two-way random-effects model with absolute agreement. ICC values and their 95% confidence intervals were reported, with ICC >0.75 indicating good agreement. Bland–Altman analysis: Bland–Altman plots were constructed to compare the measurements of the AI system and the senior group. The mean difference (bias) and 95% limits of agreement (mean ±1.96 SD) were calculated to assess systematic bias and the range of individual differences. Correlation analysis: Pearson correlation coefficients (r) were calculated between the AI measurements and the average values of the senior group, with *r* > 0.8 indicating a strong correlation.

### 2.7 Experimental platform and statistical analysis

The experimental platform for this study was built on a high-performance computing system, featuring an NVIDIA GeForce RTX 4090 GPU (24 GB VRAM), an Intel(R) Core(TM) i9-14900K processor (3.2 GHz), 32 GB RAM, and a 1.9 TB solid-state drive. The software environment was centered on PyTorch 2.5.1 for model construction and training, with OpenCV 4.10.0.84 used for image processing. Additional tools included Pandas 2.2.3, NumPy 2.0.2, Matplotlib 3.9.2, and Plotly 5.16.1 for data analysis and visualization. The entire experimental workflow was monitored and visualized in real time using Weights & Biases (wandb 0.18.7).

Model performance was comprehensively evaluated using the following metrics: sensitivity, specificity, precision, accuracy, F1 score, average precision (AP), area under the receiver operating characteristic curve (AUC), and weighted average. The calculation formulas are shown as Equations 1–8.

$$Sensitivity=\frac{TP}{TP+FN}$$ (1)

$$Specificity=\frac{TN}{TN+FP}$$ (2)

$$Precision=\frac{TP}{TP+FP}$$ (3)

$$\text{Accuracy}=\frac{TP+TN}{TP+\text{TN}+\text{FP}+\text{FN}}$$ (4)

$$F1\;Score=2\times \frac{Precision\times Sensitivity}{Precision+Sensitivity}$$ (5)

$$P_{weighted}=\sum_{i=1}^{k}w_{i}\cdot P_{i}$$ (6)

$$\text{Average }Precision\left(AP\right):\;AP=\int_{0}^{1}p\left(r\right)dr$$ (7)

$$AUC=\frac{1}{2}\sum_{i=1}^{n-1}\left(FPR_{i+1}-FPR_{i}\right)\times \left(TPR_{i+1}+TPR_{i}\right)$$ (8)

TP denotes the number of true positives, TN the number of true negatives, FP the number of false positives, and FN the number of false negatives; $P_{i}$ represents the performance metric for the i−th class, and $w_{i}$ denotes the corresponding weight of the i−th class.

## 3 Results

### 3.1 Model training and validation

**FIGURE 5:**
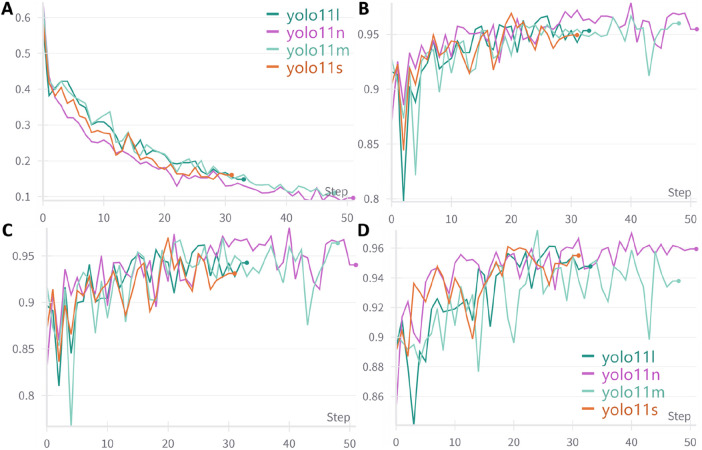
Trends in performance metrics of different models with increasing training steps. **(A)** Loss curve; **(B)** Accuracy trend; **(C)** Precision trend; **(D)** Sensitivity trend; An early stopping strategy was applied in this study, which may result in varying training step counts across different models.

A total of 4,025 images were included in this study, categorized into three classes: ileocecal part, instrument operations, and normal visible mucosa. Among them, 3,744 images were used for model development, while an independently collected set of 281 images and 48 videos was reserved for testing. Four YOLOv11 neural network models of varying scales—YOLOv11n, YOLOv11s, YOLOv11 m, and YOLOv11l—were trained on the same dataset. The entire training process was tracked using Weights & Biases (wandb). As the training steps progressed, model loss steadily decreased and eventually stabilized, indicating convergence toward optimal performance (Figure 5A). Figures 5B–D illustrate the trends of accuracy, precision, and sensitivity across different models during training. These performance metrics initially showed gradual improvement with considerable fluctuations but later stabilized at high levels. Although YOLOv11 m exhibited slightly lower sensitivity (93.79%) compared to other models, it achieved the highest accuracy (96.00%), precision (96.38%), and F1 score (94.97%), demonstrating the best overall performance. Consequently, YOLOv11 m was selected as the final model for deployment. Detailed results are presented in Table 1.

**TABLE 1:** Performance comparison of different models on the validation set (%).

Model	Accuracy%	Precision%	Sensitivity %	F1 score%
yolov11n	95.47	94.04	95.95^a^	94.92
yolov11s	94.93	93.12	95.51	94.24
yolov11 m	96.00^a^	96.38^a^	93.79	94.97^a^
yolov11l	95.33	94.27	94.77	94.51

^a^ indicates the best performance.

### 3.2 Testing and visual interpretability of the best-performing model

**FIGURE 6:**
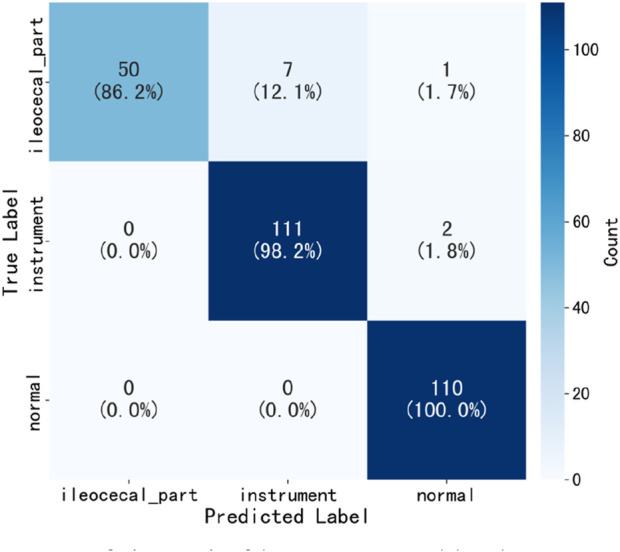
Confusion matrix of the YOLOv11 m model on the test set.

**FIGURE 7:**
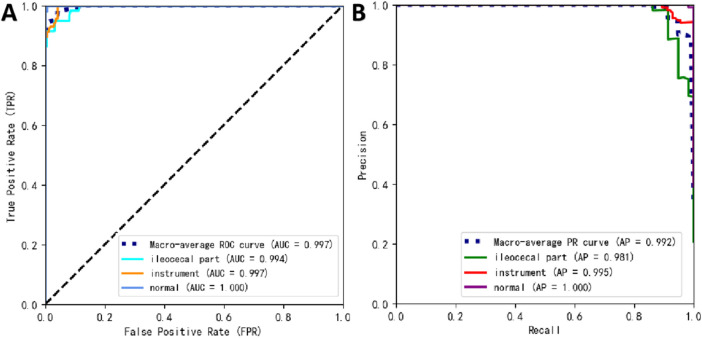
Prediction performance of YOLOv11 m on the external test set. **(A)** Receiver operating characteristic (ROC) curves; **(B)** precision–recall (PR) curves.

**FIGURE 8:**
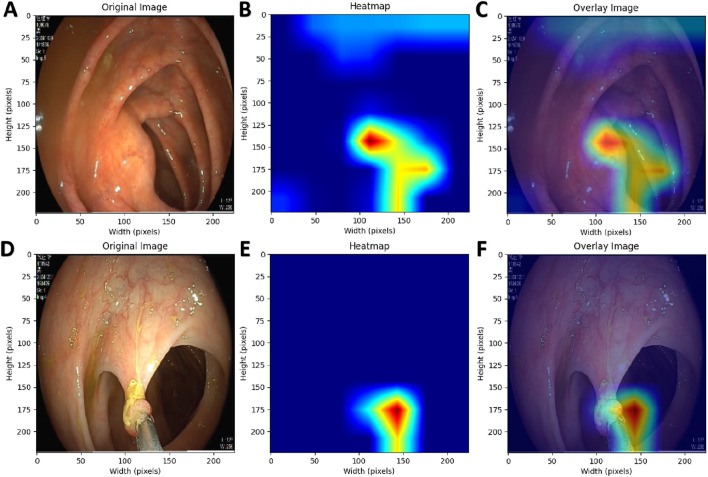
Grad-CAM Visualization of the AI Model’s Decision-Making Process. **(A,D)** Original endoscopic images; **(B,E)** Pixel activation heatmaps generated using Grad-CAM; **(C,F)** Overlay of original images and activation heatmaps.

Table 2 presents the performance of the best-performing model, YOLOv11 m, on 281 test images, including precision, sensitivity, specificity, F1 score, accuracy, average precision (AP), and AUC values for the three classes, along with a weighted average as a summary metric. Figure 6 shows the confusion matrix of the YOLOv11 m model, illustrating the distribution of predictions across all classes. The model achieved an inference speed of 86.78 FPS on the test set. Figure 7 further illustrates key evaluation curves of the YOLOv11m model: Figure 7A shows the ROC curves, with all class-specific curves closely approaching the top-left corner, indicating excellent classification performance; Figure 7B displays the precision-recall (PR) curves, where curves approaching the top-right corner reflect superior detection capabilities. Figure 8 presents Grad-CAM–based visualizations of the model's decision-making process. Figures 8A,D correspond to the ileocecal part and instrument operation categories, respectively. Figures 8B,C show activation heatmaps and overlay results for the ileocecal region, while Figures 8E,F illustrate the corresponding heatmaps and overlays for instrument operation frames. Warm-colored areas (such as red and yellow) highlight the critical lesion regions the model focused on. These visualizations help clinicians intuitively verify whether the model is focusing on medically relevant areas, which can build trust in its predictions and support clinical acceptance. For instance, in the ileocecal part category, Grad-CAM heatmaps consistently focused on the ileocecal valve—an important anatomical landmark used by endoscopists to confirm successful cecal intubation. This clear alignment between model attention and clinical reasoning further enhances trust and supports real-time clinical decisions.

**TABLE 2:** Classification performance of the YOLOv11 m model on the test set.

Class	Precision %	Sensitivity %	Specificity %	F1 score %	Accuracy %	AP %	AUC
Ileocecal part	99.97	86.21	99.96	92.59	97.15	98.13	99.39 [0.93,0.99]
Instrument	94.07	98.23	95.83	96.1	96.8	99.55	99.69 [0.94,0.99]
Normal	97.35	99.88	98.25	98.65	98.93	99.98	99.99 [0.96,1.00]
Overall (weighted average)	96.58	96.44	97.64	96.38	96.44	99.42	99.75 [0.98,0.99]

Weighted average metrics take into account the number of samples in each class, assigning greater weight to classes with larger sample sizes.

### 3.3 Application of the multitask withdrawal quality control system

**FIGURE 9:**
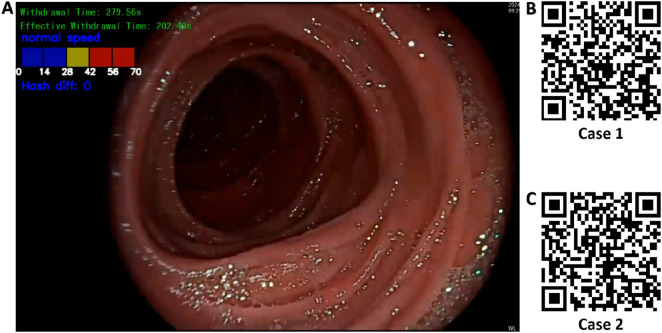
Multitask AI-Based withdrawal quality control system. **(A)** User interface of the developed multitask AI withdrawal quality control system; **(B)** & **(C)** Two example cases demonstrating colonoscopy withdrawal procedures assisted by the system.

Based on the best-performing YOLOv11 m model, this study developed a multitask colonoscopy withdrawal quality control system named EWT-SpeedNet. The system integrates image classification and speed evaluation modules to achieve three core quality control functions: automatic identification and recording of WT, automated calculation of EWT, and real-time monitoring and visual display of withdrawal speed. EWT-SpeedNet supports parallel multitask processing and provides efficient, objective, and real-time feedback without the need for manual intervention. Figure 9A shows the system’s user interface, where WT, EWT, and withdrawal speed are displayed in real time in the upper-left corner of the endoscopic video.

Case 1 (Figure 9B): During a colonoscopy procedure lasting 13 min and 35 s, once the endoscope reaches the cecum, the EWT-SpeedNet system’s WT and EWT timing modules are automatically activated. Between 5 min and 59 s and 6 min and 18 s, the patient underwent a polyp biopsy with forceps. The AI system automatically detects and subtracts the duration of this interventional procedure, enabling a more precise calculation of the EWT. Additionally, the system features an integrated visual speed scale that provides real-time feedback on withdrawal speed, helping the endoscopist maintain a controlled and appropriate withdrawal pace. With AI assistance, clinicians can focus more on mucosal observation, which may improve the detection rate of adenomatous polyps and other lesions.

Case 2 (Figure 9C): In another colonoscopy video with a total duration of 6 min and 19 s, the EWT-SpeedNet system automatically measured a WT of 2 min and 31 s and an EWT of 1 min and 55 s. During the procedure, the withdrawal speed scale repeatedly indicated in red that the withdrawal speed was in the “danger zone,” signaling that the scope was being withdrawn too quickly and that the procedure was not following standard protocols. With the support of the EWT-SpeedNet system, especially for novice or less experienced endoscopists, there is a promising potential to standardize withdrawal techniques, thereby enhancing overall examination quality and increasing the detection rate of adenomas.

### 3.4 Human–AI comparative experiment

**FIGURE 10:**
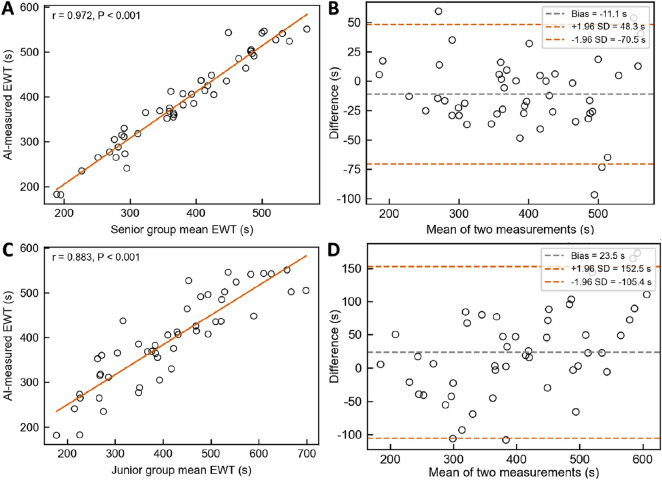
Comparison of EWT measurements between the AI System and Endoscopists of Different Experience Levels. **(A)** & **(B)** Pearson correlation analysis and Bland–Altman consistency analysis between the AI system and senior endoscopists. **(C)** & **(D)** Pearson correlation analysis and Bland–Altman consistency analysis between the AI system and junior endoscopists.

A total of 48 colonoscopy videos from the independent test set were included to evaluate the performance of the AI system in measuring effective withdrawal time (EWT) compared with endoscopists of varying experience levels, with all measurements conducted independently. The results demonstrated strong agreement between the AI system and the senior endoscopist group (ICC = 0.969, 95% CI: 0.941–0.984) and a high correlation (Figure 10A, Pearson *r* = 0.972, *p* < 0.001). However, the Wilcoxon test revealed a statistically significant difference (W = 329.5, *p* = 0.0074), and Bland–Altman analysis (Figure 10B) showed a slight underestimation by the AI system (Bias = −11.1 s, 95% LoA: −70.5 to 48.3 s),. In comparison with the junior endoscopist group, the AI system showed lower agreement (ICC = 0.838, 95% CI: 0.755–0.896) but still maintained strong correlation (Figure 10C, Pearson r = 0.883, p < 0.001); the Wilcoxon test indicated a significant difference (W = 360.0, p = 0.0187), and Bland–Altman analysis (Figure 10D) revealed an overall overestimation by the AI system (Bias = 23.5 s, 95% LoA: −105.4–152.5 s).

## 4 Discussion

Based on a colonoscopy image dataset encompassing three categories—ileocecal part, instrument operation, and normal visible mucosa—this study applied transfer learning to fine-tune four pre-trained YOLOv11 models of varying scales (nano, small, medium, and large), completing the processes of model training, validation, and testing, and ultimately selecting the best-performing model. Subsequently, the Laplacian operator was used to identify blurred frames in colonoscopy videos, and the pHash algorithm was employed to monitor withdrawal speed in real time. These components were integrated into the development of EWT-SpeedNet, an AI-assisted multitask colonoscopy withdrawal quality control system. Built on the PyTorch and OpenCV frameworks, the system operates without manual intervention and provides real-time display of key quality control indicators, including WT, EWT, and withdrawal speed, all presented within a unified visual interface. In a human–AI comparative experiment involving 48 complete colonoscopy videos, the EWT-SpeedNet system demonstrated promising clinical applicability.

In 2006, a prospective observational study by Barclay et al. (Keswani et al., 2021) demonstrated that an average WT of no less than 6 minutes significantly increased the ADR, supporting its adoption as the minimum WT standard for colonoscopy. The American Gastroenterological Association (AGA) released a clinical practice update in 2021 (Rex et al., 2024), recommending longer withdrawal times during routine colonoscopy to enhance ADR, with a suggested average WT of no less than 6 minutes and an ideal target of 9 minutes. Guidelines jointly issued by the American Society for Gastrointestinal Endoscopy (ASGE) and the American College of Gastroenterology (ACG) (Li et al., 2024) further specify that for patients aged ≥45 undergoing screening, surveillance, or diagnostic colonoscopy—without biopsy or polypectomy—the average WT should be no less than 8 minutes. In recent years, both research and clinical guidelines have increasingly emphasized quality control during the colonoscopy withdrawal phase. On one hand, they advocate for longer withdrawal times to improve ADR; on the other, they stress that interventional durations, such as those spent on biopsy or polypectomy, should be excluded from total WT, placing greater emphasis on EWT—the actual time spent inspecting the colonic mucosa. In clinical practice, “blurred frames” during withdrawal are common and unavoidable, often caused by mucosal folds obstructing the view, rapid endoscope movement, or interference from fecal fluid or residue. These frames compromise mucosal visibility and do not meaningfully contribute to lesion detection; hence, they should also be excluded from EWT calculation. To address these issues, this study developed the EWT-SpeedNet system, capable of displaying both WT and EWT in real time. It not only automatically identifies and subtracts the duration of instrumental procedures, but also accurately detects and excludes non-informative blurred frames, providing a more precise and objective tool for withdrawal quality assessment in clinical practice.

Although EWT provides a more accurate reflection of mucosal inspection quality during colonoscopy, traditional manual measurement of EWT is time-consuming, labor-intensive, and prone to subjective bias. There is an urgent need for automated, objective, and real-time monitoring enabled by technological solutions. Lui et al. (2024) proposed a novel AI-based EWT quality metric and, after analyzing 350 withdrawal videos, found a significant correlation between EWT and ADR: each additional minute of EWT increased ADR by 49% (aOR = 1.49, 95% CI: 1.36–1.65), and the AUC for predicting ADR based on EWT was significantly higher than that based on standard withdrawal time (0.80 vs 0.70, P < 0.01). Similarly, Li et al. (Rex et al., 2024) developed an EWT automatic calculation system using the YOLOv5 model, achieving a high level of agreement between AI and manual verification (r = 0.92). Unlike these prior studies, the present research innovatively introduced the Laplacian operator to quantify image sharpness by calculating the variance of the second-order derivative of grayscale values (VarL). When VarL falls below a defined threshold, the frame is classified as blurred and counted toward ineffective observation time. This method requires no additional model training and offers better interpretability and generalizability, making it more suitable for real-time deployment. Although the AI system demonstrated strong agreement with senior endoscopists in EWT measurement (ICC = 0.969, r = 0.972), Bland–Altman analysis revealed a slight underestimation (Bias = −11.1 s; 95% LoA: −70.5 to 48.3 s). However, this difference of 11.1 s is clinically acceptable, especially given that the minimum standard for effective withdrawal time in colonoscopy is typically 6 min. Such a minor deviation is unlikely to affect quality assessments or clinical decision-making. Moreover, compared with junior endoscopists, the AI system showed greater consistency with senior experts, underscoring its potential to support standardization and training in real-world practice. To reduce the underestimation bias and improve accuracy, we plan to refine the blur detection threshold and introduce post-processing calibration models based on expert annotations. Moreover, considering the interrelationship between withdrawal speed and withdrawal time, this study uniquely integrated withdrawal speed monitoring with EWT calculation within a unified system. This multitask integration not only enables real-time evaluation of EWT but also dynamically monitors withdrawal speed, effectively reducing the risk of missed lesions due to localized rapid withdrawal. Compared with previous studies, this system offers greater practicality and enhanced clinical applicability.

To further contextualize the strengths and innovations of our system, Table 3 provides a comparative summary of EWT-SpeedNet with two representative AI-based systems previously proposed by Lui et al. (2024) and De Carvalho et al. (2023). The comparison highlights major differences in task coverage, real-time capabilities, withdrawal speed monitoring, and intended clinical application.

**TABLE 3:** Comparison of EWT-SpeedNet with prior AI systems.

Feature	EWT-SpeedNet (this study)	Lui et al. (2024)	De Carvalho et al. (2023)
Tasks performed	Multitask (WT, EWT, speed monitoring)	Single-task (EWT)	Single-task (EWT)
Real-time capability	Yes	No	No
Blur frame detection	Yes (Laplacian operator)	No	No
Interventional time exclusion	Yes (AI-detected)	Yes (manual annotation)	Not mentioned
Withdrawal speed monitoring	Yes (pHash algorithm + scale bar)	No	No
Target use	Real-time quality control	Real-time quality control	Offline post-analysis

Based on the comparison with Lui et al. (2024) and De Carvalho et al. (2023).

Nevertheless, this study has certain limitations. First, our current dataset remains relatively limited for deep learning applications. To enhance model generalizability, we plan to expand the dataset through partnerships with five geographically diverse medical centers, enabling access to a broader range of clinical settings, endoscopy equipment, and patient populations. Second, we are currently preparing for a prospective, multicenter clinical trial aimed at further validating the adaptability and clinical utility of the EWT-SpeedNet system. This study will involve real-time implementation of the system in clinical workflows and assess its impact on key quality indicators such as ADR. Third, to support clinical deployment, the EWT-SpeedNet system is designed for seamless integration into existing workflows. It processes colonoscopy video in real time, overlays withdrawal speed and timing information, and provides immediate feedback to endoscopists without requiring additional hardware. Future versions will incorporate a user interface and reporting features to enhance usability and support quality control.

## 5 Conclusion

This study proposed a multitask withdrawal quality control system that integrates real-time monitoring of withdrawal time, effective withdrawal time, and withdrawal speed. The system is capable of assisting endoscopists during the colonoscopy withdrawal process by proactively prompting control over withdrawal speed and stability. It holds promise for reducing inter-operator variability and enhancing the overall quality of routine colonoscopy procedures.

## Data Availability

The raw data supporting the conclusions of this article will be made available by the authors, without undue reservation.
